# Supplementary material for: Effect of Alkali on the Microbial Community and Aroma Profile of Chinese Steamed Bread Prepared with Chinese Traditional Starter
Source: Foods. 2023 Feb 1;12(3):617. doi: 10.3390/foods12030617 (PMC9914934; doi:10.3390/foods12030617)
Supplement: Supplementary file 1 [file foods-12-00617-s001.zip › foods-2145647-supplementary.pdf]

Supplemental Materials

Table S1. Information about the strains isolated from Chinese traditional starters (CTSs) used to prepare synthetic microbial community starters.

Starter	Lactic acid bacteria		Yeast	
	Strain	Sampling position	Strain	Sampling position
PP1	*Pediococcus pentosaceus* AX0015 (OM956360)	Lankao City, Henan Province	*Saccharomyces cerevisiae* AX0002 (OM956365)	Lankao City, Henan Province
PP2	*Pediococcus pentosaceus* AX0019 (OM956361)	Lankao City, Henan Province		
CC1	*Companilactobacillus crustorum* AX0037 (OM956362)	Weinan City, Shaanxi Province		
CC2	*Companilactobacillus crustorum* AX0039 (OM956363)	Weinan City, Shaanxi Province		
WC1	*Weissella cibaria* AX0057 (ON025150)	Binzhou City, Shandong Province		
WC2	*Weissella cibaria* AX0059 (ON025151)	Binzhou City, Shandong Province		

Table S2. Diversity indexes of fungi communities and bacterial communities in Chinese traditional starters (CTSs), alkali-free fermented doughs (FDs), and alkali-added fermented doughs (AFDs) from eight regions of China.

Sample	Fungi diversity indexes			Bacterial diversity indexes		
	Shannon	Chao1	OTUs	Shannon	Chao1	OTUs
WW-S	0.004	5	45200	1.364	19	48491
WW-F	0.019	12	56440	1.245	27	49168
WW-A	0.006	6	63521	1.034	17	47473
WN-S	0.020	6	35555	0.510	31	44634
WN-F	0.027	10	52275	0.115	17	40379
WN-A	0.002	3	36534	0.525	21	38281
YC-S	0.491	7	60930	0.735	92	44596
YC-F	0.313	9	64086	0.036	14	34143
YC-A	0.103	13	44057	0.197	51	45502
LY-S	0.007	3	50694	0.974	32	43231
LY-F	0.011	9	53036	0.368	23	41481
LY-A	0.010	11	58088	0.490	18	45490
LK-S	0.334	10	43625	1.139	71	47407

LK-F	0.023	7	50092	0.679	43	40788
LK-A	0.026	7	48071	0.686	43	41868
HZ-S	0.105	7	40578	1.520	45	55287
HZ-F	0.056	9	49077	0.270	26	45230
HZ-A	0.010	7	51353	0.624	25	42029
BZ-S	0.005	3	38097	1.462	72	47961
BZ-F	0.003	10	56673	0.121	39	40205
BZ-A	0.004	10	44057	0.200	35	37401
WH-S	0.124	6	44090	1.061	23	51504
WH-F	0.049	9	55711	0.386	18	42931
WH-A	0.015	10	46108	0.422	17	46889

Note: WW-S, WN-S, YC-S, LY-S, LK-S, HZ-S, BZ-S, and WH-S represent CTSs sampled from eight regions, respectively; WW-F, WN-F, YC-F, LY-F, LK-F, HZ-F, BZ-F, and WW-F represent FDs fermented by CTSs from eight regions, respectively; WW-A, WN-A, YC-A, LY-A, LK-A, HZ-A, BZ-A, and WW-A represent AFDs fermented by CTSs from eight regions, respectively.

Table S3. Concentrations of aroma compounds in alkali-free CSBs and alkali-added CSBs fermented by CTSs from eight regions of China.

Compounds	Sym.	Odors	RI	Concentrations (mg/kg)															
				WW-FB	WW-AB	WN-FB	WN-AB	YC-FB	YC-AB	LY-FB	LY-AB	LK-FB	LK-AB	HZ-FB	HZ-AB	BZ-FB	BZ-AB	WH-FB	WH-AB
Ethanol	Acl1	Alcoholic	3.08	93.86	76.73	193.13	169.23	19.48	27.65	152.57	124.05	53.96	62.09	174.41	173.07	196.90	265.38	137.56	132.08
3-Methyl-1-butanol	Acl2	Balsamic, alcoholic, malty	8.23	8.69	5.99	16.94	14.35	2.81	1.31	15.81	9.94	9.69	8.07	16.90	11.89	16.39	18.60	12.59	9.10
1-Pentanol	Acl3	Balsamic, fruity, fusel-like sweet	9.80	0.53	0.40	0.57	0.37	0.66	1.02	0.59	1.10	1.04	1.58	0.82	1.01	0.45	0.69	0.36	1.00
1-Hexanol	Acl4	Green grass, flowery, woody	12.19	8.19	8.37	8.88	6.61	13.19	18.38	14.25	16.29	24.76	32.08	23.63	30.46	10.20	16.23	5.43	9.60
1-Octen-3-ol	Acl5	Mushroom-like	13.74	3.07	2.21	3.19	2.31	3.14	4.39	3.54	4.99	4.72	5.09	5.10	5.37	3.05	0.07	2.46	4.01
1-Heptanol	Acl6	Green	14.35	0.84	0.78	0.87	0.57	0.70	0.93	1.09	1.57	1.20	1.22	1.37	1.27	1.02	1.54	0.60	1.07
1-Octanol	Acl7	Earthy, mouldy vegetable	16.08	1.60	1.57	2.89	12.18	2.99	7.75	6.12	10.45	11.49	9.78	10.69	9.35	6.35	9.27	3.58	2.60
1-Nonanol	Acl8	Citrus	17.37	0.26	0.31	0.30	0.33	0.18	0.20	0.84	0.95	0.47	0.57	1.04	1.28	0.63	0.93	0.27	0.48
Benzyl alcohol	Acl9	Faint aromatic odor	20.24	0.11	0.10	0.23	0.23	0.12	0.18	0.22	0.21	0.24	0.22	0.20	0.24	0.20	0.23	0.16	0.20
Phenylethyl Alcohol	Acl10	Rose-honey-like, wilted rose	20.62	12.86	9.54	40.39	33.50	5.10	7.80	14.64	13.09	5.05	4.06	9.44	8.98	32.22	32.48	12.01	6.68
2-Pentylfuran	Fur1	Fruity aroma	9.31	12.49	7.42	17.93	12.09	20.27	16.08	20.91	18.30	52.28	48.87	65.01	27.48	16.62	8.23	16.78	12.07
Hexanal	Ald1	Green, grassy, tallow	5.78	7.09	2.86	3.62	2.81	4.34	4.35	3.94	23.85	7.29	9.71	5.90	9.65	2.89	6.89	3.54	26.78

Heptanal	Ald2	Fatty, rancid, citrus, malty	8.21	0.43	0.67	0.48	0.87	0.69	1.27	0.96	3.35	0.80	1.45	0.69	1.64	1.11	2.54	1.02	3.69
Octanal	Ald3	Citrus, flowery	10.70	0.24	0.12	0.36	0.87	0.30	0.38	0.67	1.31	1.07	0.71	0.32	0.33	0.47	0.60	0.73	1.46
(Z)-2-Heptenal	Ald4	Green, fatty	11.51	1.19	0.18	0.95	0.48	0.36	0.45	0.78	0.84	1.14	0.70	0.57	0.20	0.52	0.33	0.72	0.83
Nonanal	Ald5	Citrus, soapy	13.10	0.77	0.44	1.00	1.20	0.75	0.75	1.47	2.16	2.07	2.00	2.35	2.71	1.21	1.24	1.04	1.70
Benzaldehyde	Ald6	Almond, caramel	15.59	2.43	1.16	6.02	5.39	2.19	1.36	4.18	2.99	5.86	6.92	4.01	2.22	5.76	2.92	4.06	2.06
(Z)-2-Nonenal	Ald7	Fatty, tallowy, green	15.76	0.33	0.18	0.48	0.31	0.32	0.19	0.71	0.54	0.86	0.70	0.80	0.42	0.45	0.32	0.39	0.32
Phenylacetaldehyde	Ald8	Honey-like, sweet	17.40	0.20	0.09	0.74	0.35	0.37	0.31	0.48	0.28	0.89	0.37	0.27	0.12	0.24	0.16	0.24	0.12
(E,E)-2,4-Nonadienal	Ald9	Deep fat fried	18.16	0.48	0.10	0.33	0.14	0.16	0.07	0.33	0.47	0.58	0.43	0.38	0.15	0.24	0.25	0.26	0.75
(E,E)-2,4-Decadienal	Ald10	Deep fat fried, waxy	18.95	1.05	0.28	1.17	0.35	0.45	0.38	0.77	0.59	1.13	1.08	1.39	0.53	0.58	0.25	0.83	0.36
Acetic acid	Aci1	Sour, acid, pungent	14.44	13.21	1.04	8.69	3.10	1.36	0.54	8.14	0.89	6.09	1.68	5.92	1.96	2.95	0.57	3.94	0.61
Propanoic acid	Aci2	Rancid, pungent	15.91	3.03	0.23	5.49	1.67	0.17	nd	3.20	0.44	0.64	nd	0.65	nd	3.13	nd	0.77	nd
Butanoic acid	Aci3	Sweaty, rancid	17.21	8.80	0.73	21.26	6.07	1.77	0.18	8.97	0.82	1.38	0.32	2.55	nd	3.77	0.42	3.05	0.17
Pentanoic acid	Aci4	Sweaty	18.62	0.66	nd	0.89	0.28	0.20	nd	0.60	nd	0.92	0.34	0.46	nd	0.36	nd	0.56	nd
Hexanoic acid	Aci5	Sweaty, cheesy, fatty, goat-like	19.87	8.68	1.25	5.18	1.95	1.59	0.26	4.66	1.11	9.74	4.57	7.29	0.54	3.05	0.72	3.79	0.92
Heptanoic acid	Aci6	Cheese, fatty, sweaty	21.02	0.25	0.09	0.24	0.18	0.13	nd	0.21	0.11	0.29	0.21	0.21	0.18	0.21	nd	0.25	0.16
Octanoic acid	Aci7	Cheese, fatty,	22.10	1.14	0.34	0.50	0.57	0.29	0.25	0.52	0.28	0.46	0.52	0.43	0.15	0.49	0.28	0.60	0.37

		sweaty, soapy																	
Nonanoic acid	Aci8	Cheese, fatty, sweaty	23.12	0.32	0.38	0.30	0.89	0.42	0.22	0.32	0.49	0.24	0.69	0.46	0.54	0.45	0.55	0.59	0.58
Acetone	Ket1	Ether, grape	2.03	0.13	0.17	0.23	0.21	0.30	0.31	0.42	0.29	0.56	0.45	0.25	0.37	0.32	0.28	0.30	0.24
2-Heptanone	Ket2	Soapy, fruity, cinnamon	8.14	0.50	0.84	1.05	1.17	0.95	2.26	1.27	2.97	1.22	2.44	1.68	8.00	1.65	4.11	1.29	4.07
2-Octanone	Ket3	Soapy, fruity	10.60	0.46	0.58	0.38	0.41	0.39	0.59	0.82	1.29	1.85	2.47	1.29	3.21	1.01	1.35	0.78	1.28
Acetoin	Ket4	Butterscotch, butter, yogurt, cream	10.63	2.40	2.14	6.60	2.72	3.12	3.50	2.26	2.17	4.86	4.11	3.02	1.55	2.37	2.34	3.22	3.96
3-Nonanone	Ket5	Fruity	12.25	0.10	0.16	0.39	0.24	0.09	0.33	0.17	0.18	0.16	0.20	0.13	0.14	0.20	0.20	0.28	0.15
3-Octen-2-one	Ket6	Earthy type	13.39	0.39	0.23	0.55	0.48	0.39	0.49	0.68	1.04	1.29	1.63	0.71	1.07	0.63	0.95	0.39	1.39
Methyl acetate	Est1	Fragrant, fruity odor	2.10	0.10	0.11	1.08	0.83	0.53	0.63	0.58	0.68	0.71	0.94	0.53	0.83	0.71	0.71	0.10	0.25
Ethyl Acetate	Est2	Sweet, fruity, pineapple	2.54	2.42	1.92	1.81	1.91	42.88	53.52	2.76	2.91	59.10	70.97	0.75	1.40	46.05	44.98	2.08	2.96

Note: WW-FB, WN-FB, YC-FB, LY-FB, LK-FB, HZ-FB, BZ-FB, and WH-FB represent alkali-free CSBs fermented by CTSs from eight regions, respectively. WW-AB, WN-AB, YC-AB, LY-AB, LK-AB, HZ-AB, BZ-AB, and WH-AB represent alkali-added CSBs fermented by CTSs from eight regions, respectively. RI: retenion indice; nd: compounds not detected.

Table S4. Concentrations of aroma compounds in alkali-free CSBs and alkali-added CSBs fermented by synthetic microbial community starters.

Compounds	Concentrations (mg/kg)											
	CC1-FB	CC1-AB	CC2-FB	CC2-AB	WC1-FB	WC1-AB	WC2-FB	WC2-AB	PP1-FB	PP1-AB	PP2-FB	PP2-AB
Ethanol	6.37	8.73	2.59	2.39	38.50	53.21	33.92	33.24	76.49	60.76	88.00	70.18
3-Methyl-1-butanol	1.75	0.84	1.71	1.11	0.58	0.48	0.66	0.34	7.85	6.71	8.28	7.09
1-Pentanol	1.18	0.80	1.21	0.99	0.69	0.72	0.67	0.53	0.77	0.72	0.84	0.74
1-Hexanol	23.18	18.07	22.93	21.26	15.55	16.52	14.42	11.04	24.99	16.75	26.46	15.80
1-Octen-3-ol	4.33	3.87	4.24	3.85	3.47	3.65	3.03	2.28	4.52	3.64	4.61	3.50
1-Heptanol	0.76	0.88	0.74	0.85	0.72	0.88	0.62	0.58	1.12	0.97	1.13	0.86
1-Octanol	0.39	0.54	0.42	0.48	0.60	0.57	0.37	0.46	0.86	0.75	0.90	0.84
1-Nonanol	0.04	0.85	0.56	0.74	0.55	0.83	0.52	0.67	0.82	0.65	0.80	0.63
Benzyl alcohol	0.20	0.20	0.21	0.20	0.18	0.21	0.18	0.16	0.24	0.21	0.25	0.19
Phenylethyl Alcohol	0.52	0.54	0.45	0.39	0.44	0.48	0.41	0.34	2.22	1.66	2.34	1.60
2-Pentylfuran	36.13	27.09	40.51	22.30	28.92	22.36	22.38	12.67	40.91	17.75	38.01	15.50
Hexanal	6.67	5.38	7.72	6.53	4.94	4.09	4.37	3.06	7.26	4.29	7.40	4.85
Heptanal	0.53	0.78	0.62	0.82	0.59	1.00	0.46	0.66	0.57	0.66	0.59	0.70
Octanal	0.26	0.24	0.22	0.28	0.23	0.28	0.24	0.16	0.34	0.29	0.37	0.27

(Z)-2-Heptenal	1.61	1.35	1.66	1.35	0.91	0.85	0.95	0.59	0.81	0.55	0.99	0.65
Nonanal	0.98	1.48	0.97	1.24	1.14	1.11	1.23	0.76	2.75	1.94	2.74	1.69
Benzaldehyde	4.35	4.47	4.51	5.10	4.61	4.45	3.76	3.10	3.49	3.72	3.71	3.71
(Z)-2-Nonenal	0.80	0.96	0.76	0.84	0.51	0.49	0.41	0.29	1.05	0.57	1.02	0.49
Phenylacetaldehyde	0.48	0.30	0.49	0.31	0.31	0.19	0.28	0.13	0.42	0.26	0.43	0.23
(E,E)-2,4-Nonadienal	0.56	0.36	0.56	0.34	0.25	0.14	0.23	0.10	0.54	0.22	0.51	0.20
(E,E)-2,4-Decadienal	2.75	2.39	2.60	2.17	0.61	0.34	0.71	0.25	1.52	1.01	1.40	0.94
Acetic acid	9.71	10.09	9.19	9.78	17.44	7.12	14.39	5.06	5.13	4.56	6.44	5.19
Propanoic acid	0.37	0.35	0.35	0.31	0.05	0.03	0.04	0.03	0.59	0.45	0.59	0.51
Butanoic acid	1.21	0.60	0.63	1.07	0.38	0.27	0.41	0.38	0.63	0.55	0.72	0.60
Pentanoic acid	0.99	0.85	1.01	0.87	0.66	0.36	0.59	0.28	1.07	0.69	1.14	0.73
Hexanoic acid	6.76	6.46	7.13	6.22	4.86	2.87	4.40	2.22	11.87	6.08	12.23	6.03
Heptanoic acid	0.23	0.23	0.25	0.23	0.20	nd	0.16	nd	0.39	0.24	0.39	0.22
Octanoic acid	0.27	0.40	0.32	0.45	0.25	0.29	0.24	0.22	0.64	0.67	0.58	0.52
Nonanoic acid	0.40	0.34	0.46	0.39	0.30	0.39	0.31	0.26	0.41	0.45	0.43	0.41
Acetone	0.50	0.43	0.47	0.46	nd	0.29	0.20	0.24	0.38	0.38	0.50	0.51
2-Heptanone	2.38	1.66	2.22	1.86	0.69	0.85	0.64	0.72	0.96	0.79	1.00	0.89

2-Octanone	1.02	0.74	0.96	0.72	0.37	nd	nd	0.39	nd	0.85	0.91	0.86
Acetoin	4.94	2.68	4.82	3.22	3.23	5.38	5.28	5.64	4.30	3.36	4.76	3.25
3-Nonanone	0.16	0.21	0.20	0.20	0.22	0.28	0.23	0.37	0.19	0.19	0.22	0.25
3-Octen-2-one	0.70	0.71	0.66	0.69	0.70	0.68	0.56	0.39	0.65	0.45	0.57	0.37
Methyl acetate	0.64	0.76	0.11	0.14	0.58	0.83	0.14	0.18	0.49	0.56	0.10	0.22
Ethyl Acetate	50.34	59.18	17.91	20.00	40.06	56.09	39.63	49.91	42.30	51.77	18.82	36.07

Note: WC1-FB, WC2-FB, CC1-FB, CC2-FB, PP1-FB, and PP2-FB represent alkali-free CSBs fermented by WC1, WC2, CC1, CC2, PP1, and PP2 synthetic microbial community starters, respectively. WC1-AB, WC2-AB, CC1-AB, CC2-AB, PP1-AB, and PP2-AB represent WC1, WC2, CC1, CC2, PP1 and PP2 alkali-added CSBs fermented by synthetic microbial community starters, respectively.

Figure captions

Figure S1. Relative abundances of microbial communities in CTS, FD, and AFD groups at the phylum level. (A) Fungal communities. (B) Bacterial communities.

Figure S1.

A

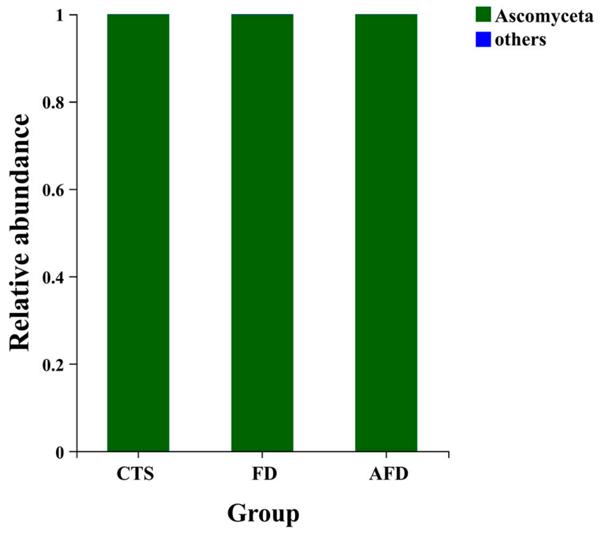

B

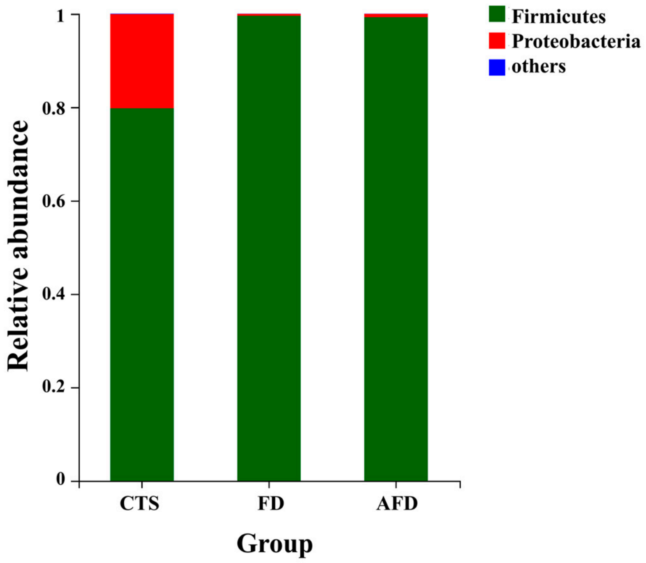

Relative abundance
1
0.8
0.6
0.4
0.2
0
CTS
FD
AFD
Group
Firmicutes
Proteobacteria
others
